# A COVID-19 pregnant patient with thrombotic thrombocytopenic purpura: a case report

**DOI:** 10.1186/s13256-020-02577-5

**Published:** 2021-03-01

**Authors:** Soheila Aminimoghaddam, Narjes Afrooz, Setare Nasiri, Ozra Motaghi Nejad, Fatemeh Mahmoudzadeh

**Affiliations:** 1 Department of Gynecology and Oncology, Iran University of Medical Sciences, Tehran, Iran; 2 Department of Emergency Medicine, Tehran University of Medical Sciences, Tehran, Iran

**Keywords:** Pregnancy, Thrombotic thrombocytopenic purpura, COVID-19, Addiction, Plasmapheresis

## Abstract

**Background:**

Pregnancy seems to increase the risk of thrombotic thrombocytopenic purpura (TTP) relapses and make the TTP more severe in any of the pregnancy trimesters, or even during the postpartum period.

**Case presentation:**

This study highlights details of treating a COVID-19 pregnant patient who survived. This 21-year addicted White woman was admitted at her 29th week and delivered a stillbirth. She was transferred to another hospital after showing signs of TTP, which was caused by a viral infection.

**Conclusion:**

This viral infection caused fever and dyspnea, and the patient was tested positive for COVID-19 infection. A chest computed tomography scan showed diffuse multiple bilateral consolidations and interlobar septal thickening. She stayed at the Intensive Care Unit for 20 days and treated with plasmapheresis. As far as we know, this is the first report of a TTP pregnant patient with COVID-19 infection.

## Introduction

Pregnancy seems to increase the risk of thrombotic thrombocytopenic purpura (TTP) relapses and make the TTP more severe in any pregnancy trimesters or even during the postpartum period [1]. TTP is diagnosed when a patient has at least three of the following symptoms: thrombocytopenia, microangiopathic hemolytic anemia (MAHA), end‑organ damage (mostly renal insufficiency), neurologic phenomena (such as seizures, strokes), and fever. It is rare to see all the symptoms in a patient [2, 3]. TTP is usually triggered by bacterial or viral infections, autoimmune diseases such as lupus, and malignancy [4].

Acquired TTP presents as a severe MAHA and thrombocytopenia in a healthy individual. In a patient with MAHA, who has a hemoglobin level lower than ten and the platelet level lower than 30,000, the patinaed will be diagnosed as a TTP patient. Lactate dehydrogenase (LDH) level and ADAMTS13 activity level can achieve the same result [5, 6]. Differential diagnoses in pregnancy involve other conditions that contribute to having MAHA. These conditions include severe preeclampsia, HELLP syndrome, and disseminated intravascular coagulation (DIC) [7].

The most important therapy for TTP is doing plasma exchange to remove anti-ADAMTS13 autoantibodies. Plasma exchange should be administrated to reach at least 150,000/mm^3^ of platelets for 3 successive days, and LDH level of at most 500 U/l [8]. Introducing this treatment was a big jump, which reduced the mortality rate of TTP to 20% compared to the previously reported 90% [9]. Glucocorticoid therapy is a second treatment for TTP patients that can reduce the creation of the anti-ADAMTS13 autoantibody [10].

This study delves into finding how COVID-19 affects the pregnancy outcome in a rare condition. It focuses on viral infections as one of the causes of TTP. We report a drug-addicted TTP patient affected by COVID-19, who just delivered her baby, and we discuss the postpartum conditions in detail. We are going to share our case to increase knowledge on how to treat TTP patients with such complicated conditions.

## Case study

A 21 years old White woman with gravida 1 and para 1 and intra uterine fetal death, who had a 29-week gestation, was admitted to RobatKarim Hospital, Tehran, Iran on March 20, 2020. The patient did not have a history of hospitalization, and she did not report any health issues among her family members. She was a housewife from a middle-class family who was living with his husband. Also, she was not a relative of her husband. She vaginally delivered a macerated male baby with 1300 g. As she stated, the pregnancy was unintended, and there was not any documented treatment found during the pregnancy months. The TTP patient who delivered her child and had a COVID-19 infection. As she stated, she did not have any severe preeclampsia signs, such as headaches and visual changes. She had fever, and sporadic dry coughs starting a week before the due date. She was tested positive for COVID 19.

She was a heavy smoker (did not take alcoholic beverages) and addicted to methamphetamine (for example, crystal) for a long time, and as she declared, she stopped taking methamphetamine during the last month before giving birth. She was treated with one loading dose of magnesium sulfate (4 g/IV (Intravenous)/stat), as well as antibiotics, such as ampicillin (2 g/IV/every 6 hours), Clindamycin (600 mg/IV/every 8 hours) and gentamicin (80 mg/IV/every 12 hours). Her condition deteriorated as the laboratory tests showed; the creatinine level became 5.6 mg/dl, the platelet was decreased to 23,000/mm^3^ and hemoglobin was decreased to 8.9 g/dl.

On March 21, 2020, she was sent to Firoozgar teaching hospital, Tehran, Iran, which Iran University of Medical Sciences administrated. At the time of admission, she was awake, conscious, and had a normal mental state. Her neurological examination, including cranial nerves, motor, sensory and cerebellar examination, were within normal ranges. Her blood pressure and body temperature and respiratory rate and pulse rate were 125/80 mmHg, 38.4 ℃ (oral), 26/minute, and 110/minute, respectively. Auscultation of both lungs and the heart showed fine crackles and tachycardia. Uterus was contracted, and we did not see any tenderness in liver palpation in the abdominal examination, and extremities examination showed that she had petechia on the inner surface of the arms.

**Fig. 1.**
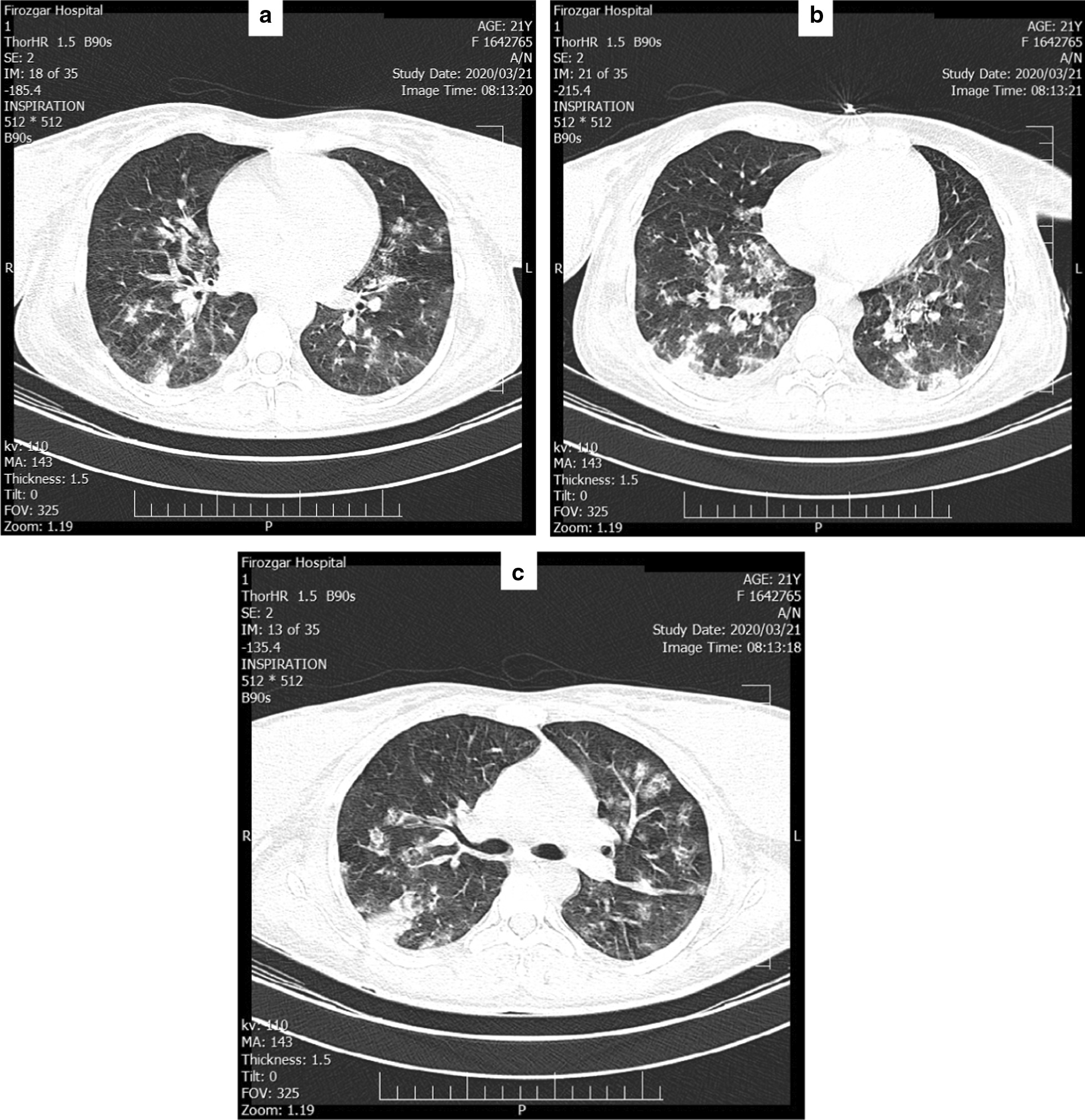
Chest computerized tomography scan of the patient; **a** Ground glass lung opacities, **b** diffuse multiple bilateral consolidations, and **c** diffuse bilateral interlobar septal thickening

The electrocardiogram was normal and Chest X-ray showed that both lungs had signs of diffused opacity. Chest computerized tomography (CT) showed signs of ground glass lung opacities (Fig. 1a), diffuse multiple bilateral consolidations (Fig. 1b), and diffuse bilateral fine interlobar septal thickening (Fig. 1c). As the literature shows, these are evidence for having viral lung infection and positive COVID-19 [11, 12].

**Fig. 2.**
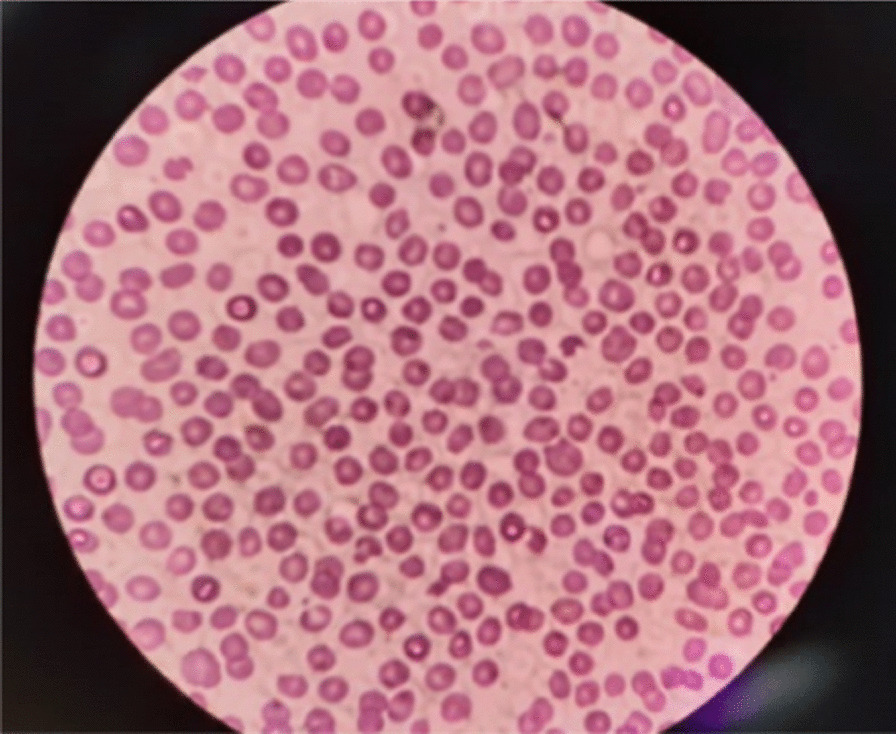
Peripheral blood smear anomalies

Figure 2 shows peripheral blood smear anomalies, including anemia, thrombocytopenia, anisocytosis, poikilocytosis, macrocytosis and schistocytes.

Polymerase chain reaction (PCR) test obtained by nasopharyngeal swabs specimen confirmed that she was positive for COVID-19. She did not have genetic testing for TTP. The viral infection tests, including Human Immunodeficiency Virus (HIV) antibody, Hepatitis B Surface (HBS) antigen, anti-HCV (Hepatitis C Virus) anti body were negative. The lupus blood test showed that anti double strand DNA, anti-coagulant, and anti-cardiolipin anti body were negative. The complement of C3 and C4 were in the normal range (C3 was equal to 101 mg/dl, and C4 was equal to 19 mg/dl).

Table 1 provides the results of laboratory tests for 5 days. As it shows for the second day, she had leukocytosis (Leukocyte was equal to 21,600 count/mm^3^), low hemoglobin level (4.9 g/dl), low platelets level (25,000/mm^3^), high serum creatinine level (4.7 mg/dl), and high lactate dehydrogenase level (1050 U/l).

**Table 1:** Laboratory results of the patient

Hospitalization day	Day 1	Day 2	Day 3	Day 4	Day5
Hemoglobin g/dl	5.0	4.9	6.1	7.9	8.1
Leukocyte count/mm^3^	11,000	21,600	29,000	28,800	18,600
Platelets /mm^3^	21,000	25,000	20,000	42,000	99,000
Fasting blood sugar mg/dl	98	87	85	87	100
Urea mg/dl	271	140	109	92	64
Creatinine mg/dl	5.6	4.7	2.3	1.3	1
Sodium mEq/l	134	143	137	142	138
Potassium mEq/l	5.7	4.7	3.6	3.4	4.2
AST u/l	24	30	33	31	36
ALT u/l	10	30	22	20	23
Bilirubin mg/dl	1.8	1.6	1.5	1.3	1.1
Albumin g/dl	2.3	3.2	3	3.5	3.7
Lactate dehydrogenase u/l	1,910	1,050	889	745	669
D-Dimer	Negative	N/A	N/A	N/A	N/A
Serum bicarbonate mmol/l	15.1	19.3	18.8	17.3	22.0
Urine analysis (protein)	Negative	N/A	N/A	1+	N/A
Urine analysis (blood)	2+	N/A	N/A	3+	N/A
Fibrinogen mg/dl	403	N/A	N/A	N/A	N/A
PT (seconds)	13.5	13	12.5	13.5	13
PTT (seconds)	24	33	25	30	28
INR	1	1.5	1.1	1.2	1.1
CRP (mg/ dl)	24	N/A	N/A	N/A	4

*AST* Aspartate Aminotransferase; *ALT* Alanine Transaminase; *PT* Prothrombin Time; *PTT* Partial Thromboplastin Time; *INR* International Normalized Ratio; *CPR* Cardiopulmonary Resuscitation; *N/A* No information was collected

Her hemoglobin level dropped to 5.0 g/dl and we transfused the packed red blood cells. Hematologists suggested taking Dexamethasone at the rate of 8 mg/day. She had premature membrane rupture along with malodor vaginal discharge and endometritis, so she took antibiotics, including Clindamycin in the rate of 600 mg/IV/(every 8 hours), Meropenem at the rate of 500 mg/(every 12 hours). It should be mentioned that the doses were adjusted to creatinine clearance. She also got dialysis treatment for 2 successive days as the serum creatinine level was raised.

Infectious disease specialists started to treat the patient with Kaletra (in one combined dosage of Lopinavir in the rate of 100 mg and Ritonavir in the rate of 400 mg) and Hydroxychloroquine 400 mg/stat followed by a continuous dose of it at the rate of 200 mg/ (every 12 hours) for14 days.

Due to the presence of thrombocytopenia and an increase in LDH level to 1910 U/l, the hematologist treated her with plasmapheresis. The hematologist suggested exchanging 2.5 l of plasma with 2 l of fresh frozen plasma for 5 consecutive days, which resulted in achieving platelet to 155,000 per cubic millimeter and lowering LDH level to 450 U/l.

Our multi-disciplinary health team strived to save the patient, and finally the patient started to feel better as the body temperature returned to normal (i.e., break the fever). The patient did not have any signs of dyspnea on the 20th day of ICU hospitalization. Additionally, the leukocyte level returned to normal, and the kidney started to function healthily. She was in ICU for 24 days until March 27, 2020, and then she was transferred to the Gynecological ward. This patient was finally discharged after 8 days. The patient is cured at the moment and does not show any TTP symptoms afterwards. Her hemoglobin is 11 g/dl, platelet is 220,000/mm^3^, LDH is 200 U/l, and creatinine is 0.9 mg/dl.

## Discussion

World Health Organization declared COVID 19 a global pandemic on March 11, 2020 [13]. COVID 19 can cause complicated situations for patients who have underlying medical conditions. A 21 years old drug-addicted woman was admitted to a hospital. She was tested positive for COVID 19 at the time of admission and delivered a stillbirth fetus. Then, she showed the early symptoms of TTP, including haemoglobin level of 5 g/dl, platelet of 23,000/mm^3^, and creatinine level of 5.6 mg/dl, and schistocytes in the peripheral blood smear. She did not have any symptoms of TTP before hospitalization. And she showed the symptoms for the first time during her pregnancy. Also, this patient had normal liver enzymes, and she did not have any severe preeclampsia symptoms. She responded to plasmapheresis and was cured. Moreover, at the moment, which is 6 months after her delivery, she does not have any TTP symptoms. By taking all these factors into account, we believe that her TTP was acquired.

Literature found that pregnant women are going through some physiological changes that affect their immune systems. This affection may predispose such people to viral respiratory infections such as COVID-19, a cause of TTP [14–16]. This study summarizes the challenges that the medical team faced during this process in order to find how COVID-19 affects the pregnancy outcome in a rare condition. As we are learning how COVID-19 interferes with organs’ functions, we share the diagnosis and treatment steps for curing a TTP pregnant woman as of the first study of its kind.

Karami *et al.*, in March 2020, reported a 27 years pregnant woman with COVID-19 symptoms such as fever, myalgia, and cough, which are similar to our case. [17] Our case has leukocytosis, which could be a positive sign for her survival; however, the 27 years case suffered from leukopenia and lymphopenia. In both of the cases, Reverse Transcription Polymerase Chain Reaction confirmed the presence of COVID-19 infection. However, the results of the Chest CT scan at the time of hospitalization were different between these studies, as the CT of our patient was similar to the CT of COVID-19 patients. Both cases started treatment for COVID 19, and both cases had a stillbirth. Literature listed Intrauterine fetal death as an outcome of infection with a family cluster of coronaviruses such as MERS (Middle Eastern Respiratory Syndrome Coronavirus)-COV and SARS(Severe Acute Respiratory Syndrome)-COV [14–16, 18]. Since we admitted our patient to the ICU sooner, she survived, while the 27 years patient died due to the multi-organ failure.

Another study took nine pregnant women with gestational ages greater than or equal to 36 weeks with COVID-19 infection, each of them had only one symptom from the typical symptoms [12] (i.e., fever, cough, myalgia, and dyspnea). Our patient was in her 29th week and had all of the common symptoms, and she survived while the patient died in this study. Each of the cases had a stillbirth, which can be explained in the case of infection. In more severe cases with COVID-19 infection, the virus may cause pneumonia that reduces the lungs’ capacity. This reduction in the capacity develops hypoxia in a pregnant patient that stops oxygen delivery to the fetus [15]. This causes Intrauterine Fetal Death (IUFD), which was happened in our case. We have a rich literature discuss the effect of influenza and other known respiratory infections in pregnancy [14], which happened to be similar in our patient who had COVID-19.

Another study considered 13 pregnant women with COVID-19; 10 cases undergone cesarean section due to the complicated conditions, such as fetal distress and premature rupture of the membrane (PROM) [15, 19]. Our case vaginally delivered a stillbirth, and the mother survived, while this study reports the death of one of the patients who had a stillbirth. This patient experienced multiple organ damages, including acute kidney injury, acute hepatic failure, and septic shock. Additionally, she went to ICU, and she was intubated due to the occurrence of acute respiratory distress syndrome [19].

Other studies treated a pregnant TTP patient effectively with plasmapheresis and glucocorticoid [20]. The major difference between this study and ours is that this study knew that the patient had TTP who finally suffered a relapse, while our patient was not previously treated for TTP. In our case, we believe that COVID-19, as a viral infection, stimulated anti-ADAMTS13 autoantibodies.

Another study treated a pregnant TTP patient who only had HELLP syndrome without COVID-19 infection [21]. The blood pressure of our case was normal, which ruled out the presence of HELLP syndrome. In both cases, high LDH, anemia, and thrombocytopenia confirm that the patients had MAHA, which is one of the hallmarks of TTP. In our case, Fig. 2 confirms this information as it visualizes fragmented erythrocytes in the shape of teardrops. To treat the TTP, our patient took Hydrocortisone 100 mg/IV/stat and then Dexamethasone 4 mg/IV/(every 8 hours) as well as having plasmapheresis daily for 5 days. It should be noted that we applied this treatment due to the severe condition of our patient, as even measuring the ADAMTS13 activities could not be suggested as a reliable diagnostic test at the acute phase [21].

## Conclusion

To summarize our case, pregnancy and the presence of the viral infection could be the triggers of TTP [6, 22, 23], which causes a critical risk for both mother and the child [24]. Literature shows that infections such as influenza, SARS-COV, and MERS-COV could increase the risk of maternal mortality, spontaneous miscarriage, preterm labor, and intrauterine growth restriction during pregnancy [15, 25]. In our case, we believe that the COVID-19 behaves similarly to other viral infections. Thus, we can consider TTP or COVID-19 as different etiologies engender the IUFD. It should be mentioned that COVID-19 does not have a proven treatment. Frontline workers are treating patients with potential therapy, such as antiviral drugs and immunotherapies. We believe that if our patient was hospitalized sooner, she could have saved the baby, as the medical procedure we followed to save this TTP patient could benefit the baby as well. We recommend to other colleagues to consider this approach while they admit a pregnant patient.

## Data Availability

Data sharing not applicable to this article as no datasets were generated or analyzed during the current study.
